# Bacterial Diversity and the Geochemical Landscape in the Southwestern Gulf of Mexico

**DOI:** 10.3389/fmicb.2018.02528

**Published:** 2018-10-18

**Authors:** E. Ernestina Godoy-Lozano, Alejandra Escobar-Zepeda, Luciana Raggi, Enrique Merino, Rosa Maria Gutierrez-Rios, Katy Juarez, Lorenzo Segovia, Alexei Fedorovish Licea-Navarro, Adolfo Gracia, Alejandro Sanchez-Flores, Liliana Pardo-Lopez

**Affiliations:** ^1^Instituto de Biotecnología, Universidad Nacional Autónoma de México, Cuernavaca, Mexico; ^2^Departamento de Innovación Biomédica, CICESE, Ensenada, Mexico; ^3^Instituto de Ciencias del Mar y Limnología, Universidad Nacional Autónoma de México, CDMX, Mexico City, Mexico

**Keywords:** bacterial community structure, amplicon taxonomic profiling, hydrocarbon adaptation, Gulf of Mexico, ocean sediments baseline

## Abstract

Marine sediments are an example of one of the most complex microbial habitats. These bacterial communities play an important role in several biogeochemical cycles in the marine ecosystem. In particular, the Gulf of Mexico has a ubiquitous concentration of hydrocarbons in its sediments, representing a very interesting niche to explore. Additionally, the Mexican government has opened its oil industry, offering several exploration and production blocks in shallow and deep water in the southwestern Gulf of Mexico (swGoM), from which there are no public results of conducted studies. Given the higher risk of large-scale oil spills, the design of contingency plans and mitigation activities before oil exploitation is of growing concern. Therefore, a bacterial taxonomic baseline profile is crucial to understanding the impact of any eventual oil spill. Here, we show a genus level taxonomic profile to elucidate the bacterial baseline, pointing out richness and relative abundance, as well as relationships with 79 abiotic parameters, in an area encompassing ∼150,000 km^2^, including a region where the exploitation of new oil wells has already been authorized. Our results describe for the first time the bacterial landscape of the swGoM, establishing a bacterial baseline “core” of 450 genera for marine sediments in this region. We can also differentiate bacterial populations from shallow and deep zones of the swGoM based on their community structure. Shallow sediments have been chronically exposed to aromatic hydrocarbons, unlike deep zones. Our results reveal that the bacterial community structure is particularly enriched with hydrocarbon-degrading bacteria in the shallow zone, where a greater aromatic hydrocarbon concentration was determined. Differences in the bacterial communities in the swGoM were also observed through a comprehensive comparative analysis relative to various marine sediment sequencing projects, including sampled sites from the Deep Water Horizon oil spill. This study in the swGoM provides clues to the bacterial population adaptation to the ubiquitous presence of hydrocarbons and reveals organisms such as *Thioprofundum* bacteria with potential applications in ecological surveillance. This resource will allow us to differentiate between natural conditions and alterations generated by oil extraction activities, which, in turn, enables us to assess the environmental impact of such activities.

## Introduction

The Gulf of Mexico (GoM) is a semienclosed sea with an area of 1,602,000 km^2^ that was formed by plate tectonics approximately 250 million years ago during the late Triassic-Jurassic (Pindell and Kennan, 2009). In the late Jurassic, the intermittent formation of fossil fuel occurred, culminating in an overload of petroleum in the terrestrial layers during the early Cretaceous (Guzman-Vega and Mello, 1999). Thus, the GoM sediments have an ancestral ubiquitous concentration of hydrocarbons maintained by two major sources: fossil fuels from natural oil seeps (MacDonald et al., 2015) and anthropogenic pollution due to the drilling, transport, and use of petroleum (Daneshgar Asl et al., 2016). The former contributes approximately 45% of the total annual hydrocarbons in the world’s oceans. In the GoM, 914 natural oil slicks have been counted, representing a discharge of approximately 10^8^ kg of oil/year (MacDonald et al., 2015). These sediment geochemical properties make the GoM a very interesting ecosystem in which to study bacterial communities and their role in this particular habitat.

Since the Deepwater Horizon (DWH) oil spill, a large number of reports describing hydrocarbon dynamics in the northern GoM has been published (Hazen et al., 2010; Kostka et al., 2011; Kappell et al., 2014; Kimes et al., 2014; Yergeau et al., 2015; Kleindienst et al., 2016). However, studies conducted before this perturbation or in the southwestern region of the GoM (swGoM) are scarce or nonexistent. Similarly, pollution levels in the deep waters of the swGoM, where 20% of the natural oil seeps are found in the GoM (MacDonald et al., 2015), have not been studied to date. This missing information is of great importance due to the near-future interest in exploration and exploitation of the swGoM region.

Deepwater marine sediments exhibit an outstanding level of uncultured bacterial diversity (Wang et al., 2012; Walsh et al., 2016). Culture-independent techniques such as 16S rRNA gene taxonomic profiling methods and high-throughput sequencing enable the study of microorganisms from natural environments. Bacteria play a very important ecological role in ocean biogeochemical processes, such as fixing and transforming carbon, nitrogen, sulfur, phosphorus, and hydrocarbons present in complex sources such as oil. In particular, sulfate is an abundant compound in the ocean (∼28 mM), and due to the presence of oil in the GoM (Orcutt et al., 2017), sulfate reduction by sulfate-reducing bacteria (SRB) is expected to happen at higher rates than in any other ocean (Bowles et al., 2011). However, this type of information concerning the swGoM is also scarce.

Environmental studies including bacterial diversity are of great interest to the evaluation and design of contingency plans and mitigation activities in case of a large-scale oil spill. Therefore, the objectives of this study are (i) to determine the first bacterial taxonomic profile in the sediments from swGoM; (ii) to compare this swGoM microbial community structure against those found in other oceans; (iii) to determine which of the measured biogeochemical factors have a higher weight in the swGoM geochemical landscape and (iv) to determine the influence of these environmental variables on the swGoM sediment bacterial community structure.

Here, we described the bacterial abundance, richness and diversity up to the genus taxonomic level that defines the bacterial fingerprint for the swGoM. The “core” genera distribution found in all swGoM sampled sites was compared against those found in five different marine sediment projects. This comparison revealed that the bacterial diversity in the swGoM is similar to that in other marine sediments, but its bacterial community structure is distinctive. The taxonomic profiles were correlated to geochemical parameters, where aromatic hydrocarbon content, depth, and organic matter were the variables with higher contributions. At some sites, significantly higher concentrations of polycyclic aromatic hydrocarbons (PAHs) were found, suggesting that hydrocarbons have the potential to shape the bacterial community structure.

Our results will allow us to differentiate between bacterial populations under primary conditions and to identify population changes that occur due to perturbations generated by oil industry activities, building the foundation for assessments of the environmental impact in the swGoM.

## Materials and Methods

### Samples Collection and Geochemical Measurements

**FIGURE 1:**
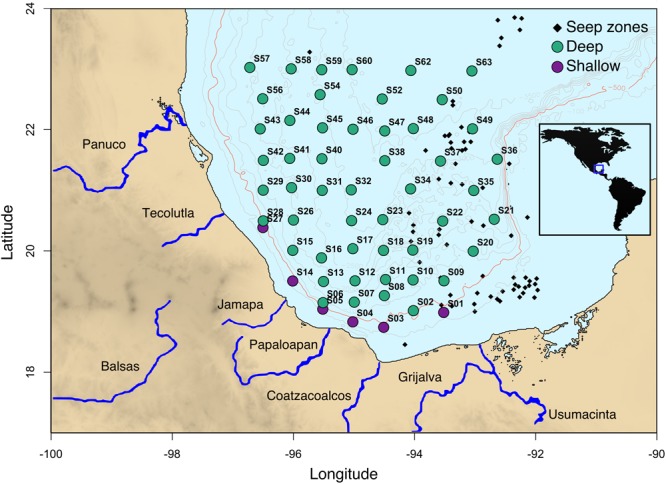
Sampling locations in the swGoM. Sediments from depths less than 500 m are shown in purple and are considered shallow. The seeps distribution depicted in black squares was compiled using (MacDonald et al., 2015), Petroleos Mexicanos (PEMEX) data and locations obtained in field surveys (unpublished data). The red line indicates 500 m depth.

In this study, a total of 56 superficial sediment samples (0–10 cm) from depths of 183 to 3740 m were collected in April 2017 onboard the R/V Justo Sierra of the UNAM using a Reineck box corer (50 × 50 cm) (**Figure 1**). This sampling event covered an area of 142,593 km^2^ in the swGoM region. We considered samples from less than 500 m depth as shallow. Sediments were stored at -70°C until processing. To plot the GoM map (**Figure 1**), we used the bathymetry database hosted by the NOAA included in the marmap library (Pante and Simon-Bouhet, 2013) in the R package (R Development Core Team, 2008).

In total, 27 aliphatic and 24 aromatic hydrocarbon compounds were extracted from the sediments with a mixed solvent solution (hexane:dichloromethane, 1:1) using an ASE 300 accelerated solvent extractor. The samples were concentrated and purified in a chromatographic column packed with sulfite, silica, alumina and granulated copper (Tolosa et al., 2004). Purified extracts were analyzed by a GC-MS system (Agilent Technologies 6890N/5973MS).

The total metals were analyzed by first digesting 0.4 g of dried sediment with 5 mL of nitric acid and 3 mL of fluorhydric acid. A second digestion was performed with 1.5 g of boric acid (Paudyn and Smith, 1992). For adsorbed metals, sediments were leached with acetic acid (0.1 M) in a synchronous rotating agitator (McLean and Bledsoe, 1992). The total and adsorbed metals (Al, V, Cr, Fe, Co, Ni, Cu, Zn, Ag, Cd, Ba, Pb, and Hg) were analyzed along with internal standards in an ICP mass spectrometer (Agilent 7500ce) and an atomic absorption spectrometer (Perkin AAnalyst 700).

Organic matter was estimated with the Walkley-Black method. Briefly, 1 mL of diphenylamine was added to the sample, and organic matter and carbon were estimated based on back titration with a 0.5 N ferrous sulfate solution (Jackson, 1958). Detailed results of all geochemical measurements are available in **Supplementary Table S1** (sheet “Abiotic Variables”).

### Sequencing and Bioinformatic Analysis

The total DNA was extracted and purified from 0.5 g of sediment using the PowerSoil DNA isolation kit (MO BIO-QIAGEN) following the recommended protocol. The DNA was used to amplify the V3-V4 16S rRNA gene variable regions using the primers S-D-Bact-0341-b-S-17 and S-D-Bact-0785-a-A-21 (Klindworth et al., 2013). Amplicon libraries were constructed as described in the 16S Metagenomic Sequencing Library Preparation protocol from Illumina and sequenced on the Illumina MiSeq platform with a paired-end read configuration of 600 cycles. Sequences are available at the SRA site under the ID SRP133184.

Reads passing the QC filters (read quality = Q20) were used to rebuild the original amplicon region (450–490 bp length) by overlapping them with Flash v1.2.7 software (Magoč and Salzberg, 2011). All nonoverlapping sequences were discarded.

Taxonomic annotation was performed using Parallel-meta pipeline v2.4.1 (Su et al., 2014) against the Metaxa2 database v2.1.1 (Bengtsson-Palme et al., 2015) as described in (Escobar-Zepeda et al., 2018). The annotation tables were formatted for MEGAN v5 (Huson et al., 2007) to generate stacked bar plots at different taxonomic levels.

The abundance matrix at the genus taxonomic level was used to calculate the Good’s coverage and alpha diversity indexes, i.e., the Chao 1 and Shannon indexes, which were estimated using the R Phyloseq library (McMurdie and Holmes, 2013). The matrix was normalized using the metagenomeSeq method (McMurdie and Holmes, 2014), and the beta diversity distance matrix was calculated using Bray-Curtis dissimilarity. A non-metric multidimensional scaling plot (NMDS) was generated through the cru.mds_meta function from the R library vegan v2.4-6 (Dixon, 2003), and a statistical analysis of the ANOSIM and ADONIS functions from the vegan R package was used to establish the existence of groups using the Bray-Curtis distance and 999 permutations. Additionally, to identify differentially abundant phylotypes in the detected sample groups, we used the fitFeatureModel function from the metagenomeSeq R library and a P-adjusted threshold value of 0.05. Fold-change charts were plotted using the ggplot2 R library (Wickham, 2016), the cladogram was generated using the ete3 (Huerta-Cepas et al., 2016) Python library, and the tree was displayed by Iroki software (Moore et al., 2017).

Abundance histograms and an absence/presence heatmap were generated using the gplots (Warnes et al., 2016), ggplot2, vegan, and Heatplus (Ploner, 2015) R libraries from the taxonomic annotation table at the genus level. The “core” corresponds to the genera present in all sites. In the shell, we included the genera with a relative abundance of >0.01% but which were absent in at least one sample, and in the cloud are genera with a relative abundance <0.01%.

### Multivariate Analysis

A multivariate principal component analysis (PCA) was performed with abiotic variables using the FactoMineR and factoextra libraries in the R package (Lê et al., 2008; Kassambara and Mundt, 2016). Additionally, to determine the weight of abiotic variables, we ran a similarity analysis and used spectral feature selection (Solorio-Fernández et al., 2017) in combination with the ReliefF method (Urbanowicz et al., 2018).

To integrate the abiotic parameters and microbial diversity at the genus level, we performed a constrained double principal coordinates analysis (cDPCoA) using the R libraries and scripts adapted from (Dray et al., 2015).

The statistical analyses used for comparison of abiotic variables (aromatic hydrocarbon, organic matter content and depth) in the shallow and deep samples groups were a Shapiro–Wilk normality test and a Mann–Whitney U test for unpaired data, both performed in R.

### Taxonomic Profiling and Diversity Comparison Study

We selected 25 sediment samples from amplicons of five projects from different oceans with the following criteria: (i) sequenced with Illumina technology; (ii) amplification of the 16S rRNA gene V3 and/or V4 variable region; and (iii) sequencing yielded = 25,000 paired-end reads. Reads were downloaded, and the taxonomic annotation was performed as described in section “Sequencing and Bioinformatic Analysis.” Venn diagrams of baseline comparisons at the genus level (genera present in all the samples from a respective group) were constructed with the Venn library of the R package (Ritchie et al., 2015) using all genera with a relative abundance >1%. Detailed information on the raw data used for comparison is available in **Supplementary Table S2**, sheet “Metadata.”

## Results

### Microbial Richness of the swGoM

As can be observed in **Figure 1**, the study area included 56 sites where sediment samples were obtained from depths ranging between 183 and 3740 m, covering an area of ∼150,000 km^2^. From the DNA sequencing data, we generated a first-glance analysis of the diversity landscape of the swGoM, using taxonomic annotation at the class level. All samples presented a homogenous pattern, and we identified 103 classes in 34 different phyla of the Bacteria domain and 4 phyla of the Archaea domain. The most abundant classes were *Gammaproteobacteria* (29.6%+/-6.0), *Deltaproteobacteria* (24.5%+/-4.7), *Alphaproteobacteria* (11.9%+/-2.0), *Clostridia* (4.6%+/-0.5), *Nitrospira* (2.9%+/-0.9), *Phycisphaera* (2.7%+/-0.5), and *Actinobacteria* (2.5%+/-0.7) (**Supplementary Figure S1**).

**FIGURE 2:**
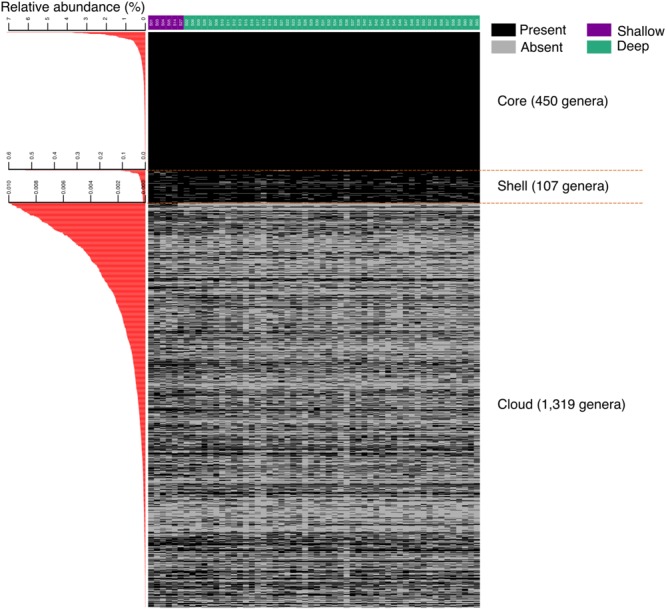
Marine sediment richness in samples from the swGoM. Core, shell and cloud groups were generated at the genus taxonomic level using an absence/presence representation. The “core” corresponds to the genera present in all sites; the “shell” includes the genera with a relative abundance >0.01% but which are absent in at least one sample; and the “cloud” corresponds to the genera with low relative abundances of <0.01%. On the left, the average of the relative abundance of each genus is shown.

In a more detailed analysis, we determined the richness of the genus per sample and observed that a group composed of 450 genera was present in all sediments. This “core” group, may be considered to define the richness baseline of the swGoM sediments (**Figure 2**). The genera defining the baseline of the swGoM are: *Thioprofundum, Rhodovibrio, Desulfovibrio, Desulfonatronum, Pelobacter, JTB255 marine benthic group, Spongiispira, Colwellia, Geoalkalibacter, Dehalogenimonas, Phycisphaera* and other aerobic or nitrate and sulfate reducers (**Supplementary Table S3**).

Additionally, we defined the “shell” group to include 107 genera not present in all samples and with abundances of up to 0.6%. Finally, the largest observed group, defined as the “cloud,” was composed of 1319 genera found at different proportions and very low relative abundances (<0.01%) in all the samples. We considered the cloud group as a part of the “rare biosphere” (Sogin et al., 2006; Lynch and Neufeld, 2015; Wang et al., 2017), although this will not be discussed in the present work (**Figure 2**).

### Comparative Taxonomic Profiling of Sediments From Several Oceans

To determine whether the swGoM bacterial taxonomic fingerprint has a particular community structure, we compared the core genera against reanalyzed sequencing data from other sediment samples around the world. We selected data from marine sediment projects collected from the North Arctic, South Pacific, South Atlantic, and South Antarctic oceans, as well as samples from the DWH spill (4–5 months after the disaster occurred) in the northeastern GoM. The analysis performed at the order taxonomic level (**Supplementary Figure S2A**), revealed differences between the swGoM samples and those from other sequencing projects. In addition to this analysis, we performed a pairwise comparison using the taxonomic profiles from each project at the genus level against the swGoM profile (**Supplementary Figure S2B**). The shared genera of sediments from different oceans and the swGoM ranged between 216 and 373, with a variable amount of genera outside the intersection.

**FIGURE 3:**
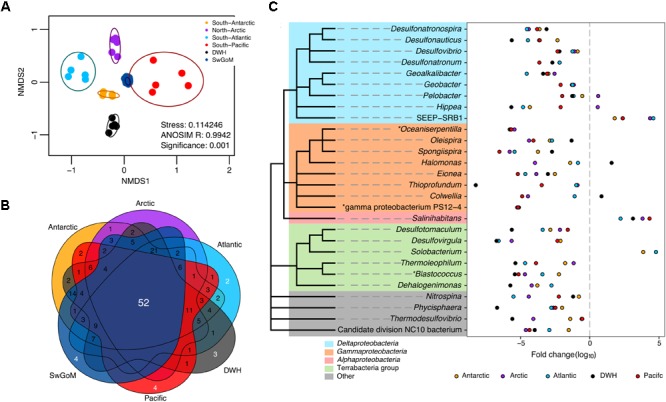
Bacterial diversity and community structure comparison between projects from different sediments in distant geographical sites. **(A)** Nonmetric multidimensional scaling plot of the Bray–Curtis beta diversity estimated from the abundance matrix at the genus level. Ellipses indicate a confidence = 0.9. **(B)** Venn diagram comparing the “core” groups of each region with the “core” of the swGoM at the genus level (>1% abundance). **(C)** Fold change of genera identified as differentially abundant in the swGoM. The asterisk mark represents unique genera in the “core” of the swGoM >1% of abundance. Negative values indicate lower abundance with respect to the swGoM and *vice versa*. Cladograms represent taxonomic association among phylotypes; but branch lengths do not reflect phylogenetic distance.

To compare the community structure and diversity of all evaluated sediments, we performed a NMDS as depicted in **Figure 3A**. Sediments from the northern GoM, corresponding to sampling 4–5 months after the DWH spill, and swGoM samples had different distribution patterns (**Figure 3A**). The swGoM baseline shares 52 genera with all sediment samples used in this comparison (**Figure 3B** and **Supplementary Table S2**, sheet “52 common all sediments BL”). Such genera mainly belong to *Deltaproteobacteria* and *Gammaproteobacteria* classes with relative abundances of >1%. Within the *Deltaproteobacteria* class, three orders contained higher genera richness—*Desulfobacterales* (8 genera), *Desulfuromonadales* (6 genera), and *Desulfurovibrionales*. The latter two were particularly abundant in the swGoM in relation to the samples from the rest of the compared sites (**Supplementary Figure S2B**). These results suggest that the swGoM diversity is similar to that found in other marine sediments but has a distinctive bacterial community structure.

We also detected phylotypes with differentially increased abundances in the swGoM in comparison to sediments from other geographical points (**Figure 3C**). Notably, several of the phylotypes have important roles in sulfur metabolism, as has been reported elsewhere. *Desulfonauticus, Desulfovibrio, Desulfonatronum, Desulfotomaculum, Desulfovirgula*, and *Thermodesulfovibrio* are SRB; *Desulfonatronospira* is a sulfidogenic organism; and *Thioprofundum* is a sulfur oxidizer. Interestingly, the genus *Thioprofundum* was one of the most abundant in all sediments except in the DWH group, from which it was absent. Finally, four genera seemed to be particular to the swGoM: *Oceaniserpentilla*, gamma proteobacterium PS12-4, *Blastococcus* and *Methylohalobius.*

### Geochemical Characterization of swGoM Sediments

**FIGURE 4:**
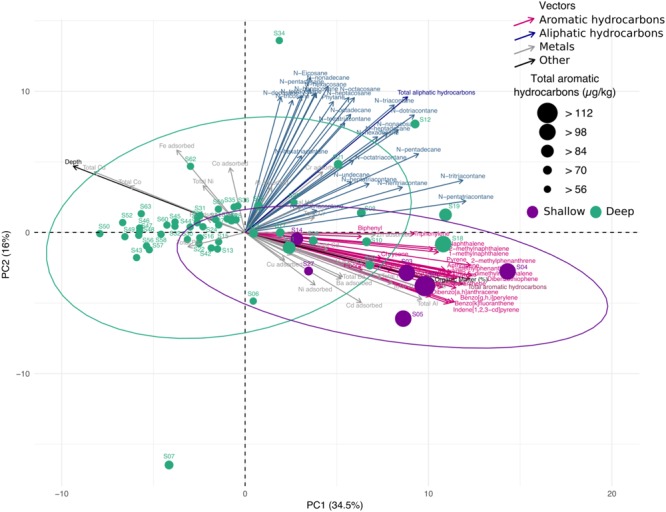
Principal component analysis of abiotic variables measured in sediment samples from the swGoM. Ellipses indicate confidence = 0.9 in groups according to depth. Circle size represents the total aromatic hydrocarbon concentrations. Sample sediments from depths less than 500 m are shown in purple.

In addition to the taxonomic profile, diversity and community structure of the bacterial population in the swGoM, we analyzed several environmental factors and measured their contribution to each sampled site. Concentrations of 53 hydrocarbons and 26 metals (total and absorbed) and organic matter contents were determined, and their contribution to the sample distribution was evaluated using a PCA (**Figure 4**). We also ranked the contributions of the measured abiotic variables using a supervised selection analysis (see “Materials and Methods”). The 10 highest-ranked variables in decreasing order were: total aromatic hydrocarbons, depth, organic matter, total Al, Zn. and Ag, Cu adsorbed, total Cu and total Co (**Supplementary Table S1**, sheet “Spectral Feature Selection”). These variables were used for further analysis of correlation with genera diversity.

Sample distribution patterns were calculated from the weight and ranking analysis of the most influential abiotic variables. Samples belonging to the shallow group formed a separate cluster, wherein the samples were primarily distributed among the total aromatic hydrocarbon vector, together with metal measurements, depth and organic matter. The two abiotic variables with higher contributions to data distribution in **Figure 4** were aromatic hydrocarbons and depth. For both shallow and deep data groups, the existence of significantly different concentrations of aromatic hydrocarbons, depth and organic matter was corroborated (**Supplementary Figure S3**). These results define the geochemical landscape in sediments from the swGoM.

Two clusters were formed by: (1) sites with depths greater than 500 m (deep group) and (2) upper-slope samples (shallow group) with depths lower than 500 m, the latter of which was the more dispersed group (**Supplementary Figure S4**). Additionally, as observed in **Figure 4**, almost all shallow sites presented a PAH concentration higher than 90 μg/kg, in contrast to the deep sites. Although the total hydrocarbon geochemical profile was homogeneous in the swGoM, the shallow region had a distinctive PAH content (**Supplementary Figure S3A**).

### The Abiotic Parameters Influence the Structure of the Microbial Population of the swGoM

To explore how the observed community structure in the swGoM is defined by the measured geochemical variables found in the sediments, we investigated the probable link between ubiquitous hydrocarbon concentrations and the abundance and distribution of bacterial genera. We started by analyzing the alpha diversity indexes and sequencing yields (**Supplementary Table S4**). The average richness of the sediment samples was 1022 ± 75 unique genera, and the average Chao1 index was 1186 ± 91. Based on the Chao1 index, the sampling effort was sufficient to reveal between 81 and 91% of the expected genera richness and coverage between 0.9974 and 0.9989. The bacterial diversity of all samples was calculated using the Shannon index value, where we found values within the range of 4.57–5.27 (including the reanalyzed data) (**Supplementary Table S2**, sheet “Metadata”).

To explain the variation in the taxonomy diversity in the context of the environmental variables, we used a cDPCoA (**Supplementary Figure S4**). In particular, we used the depth and PAH concentration to deconstruct the genera variability found in the swGoM. We found that the distinctive bacterial community structure of shallow sites can be related to the PAH content, considering the depth variation. This observation is in accordance with our previous results related to this region. To further support these results, we performed an analysis of variance (ANOVA) of reported shallow and deep sites, wherein groups are also distinguishable in terms of bacterial diversity at the genus taxonomic level (*R* = 0.8002, significance = 0.001).

**FIGURE 5:**
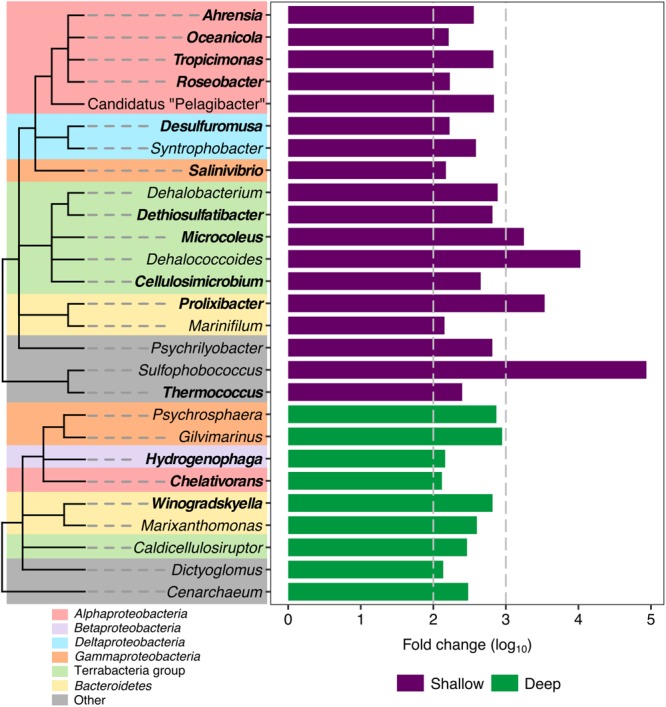
Differentially abundant genera in shallow and deep samples from the swGoM. Bars represent the fold change abundance between deep (green) and shallow (purple) samples. Genera involved in hydrocarbon degradation are labeled in bold. Cladograms represent taxonomic associations among phylotypes; but branch lengths do not reflect phylogenetic distance.

Once we confirmed the existence of shallow and deep sample groups considering the two highest-ranked variables (depth and PAH), the next objective was to define the presence of distinctive phylotypes in each group. Analysis of the differentially abundant taxa of shallow- and deep-group samples revealed an enrichment of hydrocarbon degradation genera, with 14 being more abundant in the shallow group: *Prolixibacter, Microcoleus, Tropicimonas, Dethiosulfatibacter, Cellulosimicrobium, Ahrensia, Thermococcus, Roseobacter, Desulfuromusa, Oceanicola, Salinivibrio*, and in which only three genera related to hydrocarbon degradation had a greater abundance in the deep samples: *Chelativorans, Hydrogenophaga, Winogradskyella* (**Figure 5**). Notably the, *Dehalococcoides* and *Dehalobacterium* genera, reported as halogenated compound degraders, were significantly more abundant in the shallow group samples.

## Discussion

### Bacterial Diversity From swGoM Sediments

Bacteria play a very important ecological role in ocean biogeochemical processes, such as the fixing and transformation of carbon, nitrogen, sulfur, phosphorus, etc. In the swGoM, we found a high bacterial diversity driving the biogeochemical processes expected for deep-sea sediments (Bienhold et al., 2016).

The main goal in this article is to provide the first report of a taxonomic baseline profile for bacteria inhabiting the deep-sea marine sediments of the swGoM with the highest possible resolution. The study covers an ∼150,000 km^2^ area including a group of 56 sediment samples, from which bacterial DNA was extracted and its taxonomic profile was determined up to the genus taxonomic level. A group of “core” genera can be observed in the swGoM sediments (**Supplementary Table S3**). We propose that this fingerprint could be considered as a reference baseline that describes the bacterial richness of unperturbed sediments with ubiquitous hydrocarbon concentrations. Importantly, the hydrocarbon concentrations do not correspond to those found in sediments where an oil-spill contamination has occurred. Therefore, we are describing the microbial populations adapted to particular geochemical characteristics of the swGoM.

**FIGURE 6:**
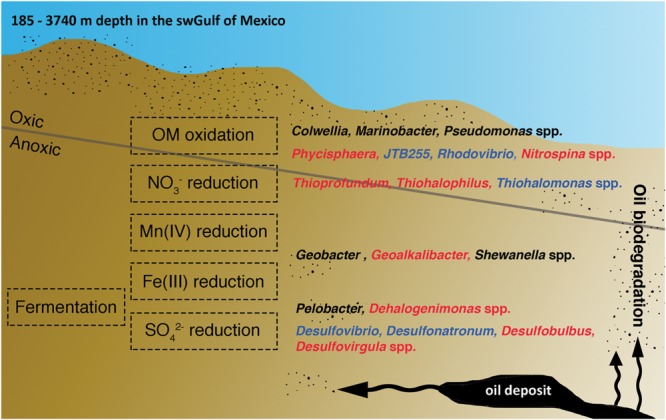
Hypothetical summary of the niche with the most abundant genera in the swGoM according to electron acceptor stratification. Blue indicates the genera that are common to marine sediments. Red and black indicate genera characteristic of the swGoM, and black indicates potential HDB.

The most abundant genera in the swGoM baseline were related to nitrate- and SRB, but the exact role of the species in the “core” and “shell” groups or low-abundant genera (“cloud” group) in the swGoM geochemical cycle remain unknown and need to be further investigated. In **Figure 6**, we show genera adapted to fit through the aerobic layer and the anaerobic NO_3_^-^, Mn (IV), Fe (III), and SO_4_^2-^ reduction strata, suggesting that all of these strata are present in sediments within a depth of 0–50 cm. Oxygen depletion is expected to occur rapidly at shelf depth but not in typical abyssal sediments (Orcutt et al., 2011), perhaps due to the continuous presence of oil at all levels and the occurrence of oil biodegradation in the GoM in both the oxic and anoxic layers.

The genera observed with high richness in the swGoM samples can be attributed to low-abundance microorganisms that are part of the “cloud” genera group (**Figure 2**). In some cases, their variable presence may be due to detection limitations related to the sequencing yield or to differential proliferation related to the physicochemical characteristics associated with each sampling point. Nevertheless, the sequencing yield used here was above that recommended to detect all dominant and subdominant taxa (Caporaso et al., 2010; Ni et al., 2017), but the sampling effort for each sample was enough to cover ∼99% of the probable sample richness.

It was interesting to identify the specific abundance of genera related to potential hydrocarbon-degrading bacteria (HDB) (Head et al., 2006; Prince, 2010). Some of these genera are commonly found in marine sediments (**Supplementary Table S3**), such as *Colwellia, Pelobacter, Geobacter, Pseudomonas, Marinobacter, Shewanella, Parvibaculum* and *Planctomyces*. Although not generally considered HDB, the last two genera mentioned have been reported to possess hydrocarbon degradation capacity or other related metabolic activities (Hilyard et al., 2008; Rosario-Passapera et al., 2012). One of the most abundant genera in the swGoM samples is *Pelobacter*, an anaerobic organism which has been isolated from marine or freshwater sediments (Schink, 2006), sewage sludge (Schink, 1984) and soils (Chauhan and Ogram, 2006). This genus plays an important role in iron- and sulfur-reducing anaerobic processes but has also been found in hydrocarbon-containing environments (Schink, 1984, 1985). The high relative abundance of *Pelobacter* in swGoM sediments may be associated with the degradation of hydrocarbons in their natural environment.

As suggested previously, potential HDB may play an important role in the hydrocarbon abundance equilibrium maintained in the swGoM. *In situ* rates of hydrocarbon degradation and several sampling periods in shorter time frames are necessary to confirm this hypothesis.

### Taxonomic Profile Comparison of Marine Sediments Around the World

After defining the bacterial fingerprint in the swGoM, we wanted to evaluate the uniqueness of this taxonomic profile and community structure. Therefore, we compared our results against those of other marine sediment projects, reanalyzing their public available data. The most abundant classes observed in the swGoM—namely, *Gammaproteobacteria, Deltaproteobacteria* and *Alphaproteobacteria*—have been previously reported as highly abundant in oxic marine sediment communities, e.g., from the Antarctic (Bowman and McCuaig, 2003), the East Pacific (Dang et al., 2008), the Eastern Mediterranean (Heijs et al., 2008), Creta (Polymenakou et al., 2009), the Pacific Arctic Ocean (Heijs et al., 2008; Li et al., 2009), the South Atlantic Ocean (Schauer et al., 2010), the South Pacific (Walsh et al., 2016), and open ocean seafloor sediments (Bienhold et al., 2016). The comparison revealed that the bacterial fingerprint in the swGoM sediments is indeed distinctive (**Figure 3A**) based on its diversity, despite having similar richnesses of the most abundant phylotypes to those of other marine sediments. As observed in our results above, the bacterial community is, in part, shaped by the hydrocarbon content.

Interestingly, in the baseline comparison at the genus level, we observed that only four genera were not shared with other sites, confirming that marine sediments have similar compositions, but the abundance of each taxa varies according to environmental factors defining the structure of the microbial community (**Figures 3A,B**). Extracting the “core” group common to all samples resulted in the identification of 52 highly abundant genera, regardless of their geographical origin (**Figure 3B** and **Supplementary Table S2**, sheet “52 common all sediments BL”). The top 10 genera based on their average relative abundances were: *Rhodovibrio, Pelobacter*, JTB255 marine benthic group, *Desulfovibrio, Desulfonatronum, Colwellia, Haliea, Geoalkalibacter, Desulfobulbus*, and *Pseudomonas*.

Sulfate-reducing bacteria are present in high abundance in all seas, since SO_4_ is the main sulfur source in ocean waters (Leloup et al., 2009). Therefore, SRB such as *Desulfonatronospira, Desulfonauticus, Desulfovibrio, Desulfonatronum*, and *Desulfovirgula* are abundant in swGoM samples (**Figure 3C**) and might play an important role in the cycling of sulfur (Barton and Fauque, 2009). The *Desulfobacteraceae* family *(Desulfosarcina-Desulfococcus* clade*)* has been recently linked to hydrocarbon degradation (Kniemeyer et al., 2007; Jaekel et al., 2015); however, other abundant genera in the swGoM such as *Desulfovibrio* (**Figure 3C**) could also participate in hydrocarbon oxidation (Kim et al., 1990; Fukui et al., 1999), which would satisfactorily explain the high sulfate reduction rates found in the GoM (Bowles et al., 2011). Other *Deltaproteobacteria* present in the swGoM are *Geobacter, Geoalkalibacter*, and *Pelobacter*, which are anaerobic bacteria capable of reducing metals (Fe and Mn). These bacteria are abundant and have been associated with the presence of hydrocarbons and their degradation, as described in other works concerning *Geobacter* (Greene et al., 2009; Musat et al., 2010).

The endemic genera present in all swGoM sediments (**Figure 3B**) but absent in samples from other oceans are (i) *Oceaniserpentilla*, which is observed in high abundance in DWH studies and is a potential cycloalkane oxidizer (Mason et al., 2012; Kleindienst et al., 2016); (ii) gamma proteobacterium PS12-4, a psychrophilic bacteria isolated from marine deep sediments (only published in genomic databases); (iii) *Blastococcus*, an Actinobacteria of the *Geodermatophilaceae* family associated with the surface of ruins, plants, and beach sediments but generally present in soil microbiota and potentially resistant to the presence of heavy metals (Chouaia et al., 2012; Pereira et al., 2015); and (iv) *Methylohalobius*, a *Gammaproteobacteria* which contains a unique species, *M. crimeensis*, a methanotrophic aerobic bacterium isolated from a hypersaline lake (Heyer, 2005).

Based on the finding of oil- and metal-resistant bacteria, all genera mentioned above are capable not only of degrading hydrocarbons but also of degrading methane and transforming heavy metals, reflecting the great biotechnological potential of the bacterial communities.

It should be noted that *Thioprofundum*, the most abundant sediment genus, was absent in all DWH samples. This genus has a versatile metabolism as a sulfur-oxidizer and organic matter degrader under different pressure and oxygen conditions (Takai et al., 2009). Additionally, other reports describing the microbial diversity in oil spill-perturbed sediments in the GoM have also either failed to observe *Thioprofundum* (Kimes et al., 2013; Lamendella et al., 2014) or found it in small proportions (<0.35%) (Kostka et al., 2011). We found *Thioprofundum* in high abundance (6.58% + /-1.52) in both shallow and deep samples in the swGoM, where the hydrocarbon concentrations do not correspond to those found in sediments overly disturbed by an oil spill.

The aforementioned features support our idea that not only the presence but also the increase in the abundance of the *Thioprofundum* genus can be considered a biomarker of health for marine sediments. However, demonstrating its vulnerability to hydrocarbons present in oil and verifying its presence in bacterial consortia tested in marine sediment health should be a next step for future work.

The most abundant genera in the DWH baseline were *Haliea, Reinekea, Colwellia, Fodinicurvata, Rhodovulum, Thiohalomonas, Pseudomonas, Thiohalophilus* and *Rhodovibrio*. All of these genera are present in the swGoM but at lower relative abundances (**Supplementary Table S3**). This suggests that the swGoM has a basal level of oil HDB that can grow to face large-scale oil spills.

### Biogeochemical Profile of swGoM Marine Sediments

To geochemically characterize the surface sediments from 56 sites in the swGoM, we measured the concentrations of 27 aliphatic and 24 aromatic hydrocarbons, 13 heavy metals and metals adsorbed to sediments and the organic matter content. We observed in most of the samples (=500 m depth) a range of PAH between 13 and 60 μg/kg, a characteristic of geographical locations distant from a contamination source (Baumard et al., 1998; de Mora et al., 2010). However, notably higher concentrations of PAHs were detected in some upper-slope samples (>100 μg/kg and <500 μg/kg). These aromatic hydrocarbon values match the range recorded in sediments of the continental platform off Campeche (16–953 μg/kg), where the highest Mexican oil extraction occurs (Gracia, 2010), and those of other oil exploitation areas in the GoM (Wade et al., 1989, 2008). Additionally, these concentrations are similar to those in marine sediments from eight countries belonging to the Gulf of Oman and the Persian Gulf, which are considered moderately polluted sites (de Mora et al., 2010; Ranjbar Jafarabadi et al., 2017).

We found that from all geochemical variables, the total concentration of aromatic hydrocarbons and depth had the highest contributions in determining the specific patterns of sampled sites of all geochemical variables (**Supplementary Table S1**, sheet “Spectral Feature Selection”). The specific influence of these abiotic variables in shallower samples results in their separation from the rest of the sites (**Figure 4**), and the specific contribution of aromatic hydrocarbon concentration and its negative correlation with depth were corroborated (**Supplementary Figure S4**). This suggests that the bacterial communities respond to environmental changes, nutrients or contaminants such as aromatic hydrocarbons.

### Environmental Variables Shape the Microbial Community

Our results indicate that microbial communities in superficial seafloor sediments are adapted to the constant presence of hydrocarbon-derived substrates and respond to the particular conditions of the swGoM. Significantly higher concentrations of aromatic hydrocarbons with respect to sediments from deeper sites (**Supplementary Figure S3A**) are present in shallower samples, where there is a chronic exposition of PAH. As previously reported (Liu and Liu, 2013), HDB become active whenever the hydrocarbon concentration increases, and this is followed by a halt in activity when concentrations decrease, reflected in a clear succession of bacterial communities. This observation is consistent with our results. However, HDB abundances in the shallow group are not within the bacterial abundance found in strongly polluted marine sediments (Yakimov et al., 2007). In spite of this, we detected the presence of HDB genera in the swGoM that could contribute to hydrocarbon catabolism in these conditions, and its abundance could increase in case of an oil spill. This is the case for *Colwellia* (2.17%), *Pseudomonas* (1.56%), *Oleispira* (0.51%), and *Alcanivorax* (0.20%).

The PAH data obtained during the 10-year surveillance in the southwest region suggest important contributions from anthropogenic inputs and, possibly, nearby natural oil seeps (**Figure 1**). These coastal sites (S1, S3, S4, S5, S14, and S27) presented differential contents of aromatic hydrocarbons (**Supplementary Figure S3A**), resulting in a separate cluster that we identified as the shallow group (**Figure 4**). The microbial diversity of this group is shaped by both PAH concentration and depth (**Supplementary Figure S4**).

Interestingly, most of the phylotypes detected as differentially abundant in the shallow group are related to hydrocarbon degradation, such as *Microcoleus* (Sánchez et al., 2005), *Ahrensia* (Gontikaki et al., 2018), and *Thermococcus* (Mardanov et al., 2009), and more specific genera such as *Tropicimonas* (Harwati et al., 2009), *Dethiosulfatibacter* (Muangchinda et al., 2013), *Cellulosimicrobium* (Qin et al., 2018), *Roseobacter* (Liu et al., 2016), *Prolixibacter* (Li et al., 2012), *Desulfuromusa* (Ramos et al., 2013), *Oceanicola* (Hassanshahian and Boroujeni, 2016), and *Salinivibrio* (Selvarajan et al., 2017) are involved in aromatic hydrocarbons degradation, supporting our hypothesis that this abiotic factor modulates the bacterial communities in the shallow region. We also found other genera such as *Psychrilyobacter*, which participates in the anaerobic degradation of organic matter (Graue et al., 2012), and *Dehalococcoides* (Nuzzo et al., 2017) and *Dehalobacterium* (Yimiti et al., 2011), which detoxify anoxic contamination in marine sediments, such as polychlorinated biphenyls.

It is worth mentioning that coastal regions of swGoM have a constant effluent of pollutants from the Tonalá, Coatzacoalcos, Papaloapan and Grijalva/Usumacinta Rivers, which are moderately polluted due to oil transport activities and the establishment of petrochemical plants (Gracia et al., 2005; Ponce-Vélez, 2005). Furthermore, the hydrocarbon industry activity near the Coatzacoalcos river makes both the riverbank and its mouth highly vulnerable to crude oil spills (Mendoza-Cantú et al., 2011; Ruiz-Fernández et al., 2016). The presence of PAHs in particular has been reported due to oil spills, as well as pyrogenic activity on petrochemical wastes. Additionally, PAH measurements in shallow and deep sediments have been used to evaluate the risk and assess the health of the ecosystem due to their greater persistence in the environment (Barakat et al., 2011; Wang et al., 2014; Adhikari et al., 2016; Ranjbar Jafarabadi et al., 2017).

## Conclusion and Perspectives

We report for the first time the baseline bacterial taxonomic profile in swGoM sediments up to the genus taxonomic level. To our knowledge, there is no previously existing information at this resolution, and this study will be a valuable contribution to the microbial ecology of a very particular niche such as the swGoM.

The uniqueness of this bacterial fingerprint was confirmed by comparison with sediments from distant geographical points in the world, where the richness of the most abundant phylotypes is similar but the community structure varies. The taxonomic profile of swGoM sediments is enriched in bacterial genera such *Oceaniserpentilla*, gamma proteobacterium PS12-4, *Blastococcus* and *Methylohalobius*, which are distinctive in this region. In addition, bacteria of the order *Desulfobacterales* and, particularly, SRB genera are also abundant. Interestingly, some of these genera have been reported as hydrocarbon degraders. Furthermore, differential abundances of potential PAH-degrading bacteria were found in the shallow samples in relation to the deeper swGoM samples.

Analysis of the abiotic variables showed that the geochemical profile was homogeneous in the swGoM. However, there were sites near the shore (shallow region) with distinctive hydrocarbon contents. These results called our attention to PAH compounds, which also had the highest contribution.

Particularly, genera related to hydrocarbons degradation such as *Prolixibacter, Microcoleus, Tropicimonas, Dethiosulfatibacter, Cellulosimicrobium, Ahrensia, Thermococcus, Roseobacter, Desulfuromusa, Oceanicola*, and *Salinivibrio* were found in shallow water sediments, and *Chelativorans, Hydrogenophaga*, and *Winogradskyella* were found in the deep depths. Furthermore, we detected the presence of genera highly recognized as HDB with considerable abundance in the swGoM. This suggests that the hydrocarbon-degrading bacteria are at basal levels in the swGoM and can increase their growth to contend with a large-scale oil spill. Interestingly, the genus *Thioprofundum* was found in all marine sediments from all over the world, with the exception of DWH samples. Therefore, we propose this genus as a health biomarker for marine sediments, which decreases its abundance under hydrocarbon pollution conditions.

Our results confirmed that most sites with a higher PAH concentration presented a different community structure within the swGoM, suggesting that hydrocarbons can shape the structure of the bacterial community. We also found that despite the ubiquitous hydrocarbon content in the swGoM, the bacterial fingerprint is not similar to that found in sediments contaminated with hydrocarbons, such as those from the DWH oil spill. This is an important characteristic, since the relationship found between the bacterial diversity and the chronic discharge of the concentration of fossil fuels present in the swGoM allows us to differentiate between changes in bacterial diversity due to alterations such as oil extraction activities that usually occur near the coast and acute disturbances such as the DWH oil spill. Additional work using whole metagenome shotgun sequencing analysis could reveal the metabolic potential of the observed bacterial fingerprint and allow us to determine the genes and their products involved directly with the hydrocarbon profile found in a community structure with a PAH chronic exposure, one disturbed by an oil spill and one with no hydrocarbon perturbations.

Taxonomic profiling studies are snapshots of bacterial communities in a particular environment and at a certain time point, and their population dynamics are determined by interactions and variables that are not yet well understood. This work provides the first indications that the ubiquitous but variable presence of hydrocarbons in the swGoM results in the adaptation of certain bacterial populations and influences bacterial diversity and select populations.

Finally, this research is part of a joint effort to study the Gulf of Mexico, founded in 2015 as a scientific research consortium^1^. Our results will contribute to the construction of a large database with information about the bacterial community found in sediments and water samples. This resource will allow us to establish contingency plans and foresee mitigation activities in the event of large-scale oil spills in the Gulf of Mexico, as well as generate information that allows the evaluation of their environmental impact.

## Author Contributions

AS-F, AG, and LP-L conceived and designed the experiments. EG-L, AE-Z, and LR performed the experiments and analyzed the data. EG-L, AE-Z, LR, AG, AS-F, and LP-L wrote the manuscript and prepared the figures and tables. AL-N managed the L4-CIGOM consortium resources and guaranteed their availability to perform the experiments and analysis. LP-L coordinated the IBT-L4-CIGOM group. AG coordinated the sampling of the oceanographic campaign and the quantification of abiotic variables. EG-L, AE-Z, LR, EM, RG-R, KJ, LS, AL-N, AG, AS-F, and LP-L contributed to the improvement of the project and reviewed the final version of the manuscript.

## Conflict of Interest Statement

The authors declare that the research was conducted in the absence of any commercial or financial relationships that could be construed as a potential conflict of interest.

## Footnotes

1 http://www.cigom.info/
