# Supplementary material for: Bacterial Diversity and the Geochemical Landscape in the Southwestern Gulf of Mexico
Source: Front Microbiol. 2018 Oct 18;9:2528. doi: 10.3389/fmicb.2018.02528 (PMC6200919; doi:10.3389/fmicb.2018.02528)
Supplement: Supplementary file 5 [file Data_Sheet_1.docx]

***Supplementary Material***

Bacterial diversity and the geochemical landscape in the southwestern Gulf of Mexico

Godoy-Lozano E. Ernestina^1+^, Escobar-Zepeda Alejandra^1+^, Raggi Luciana^1,4+^, Merino Enrique^1^, Gutiérrez-Rios Rosa Maria^1^, Juarez Katy^1^, Segovia Lorenzo^1^, Licea-Navarro Alexei F.^2^, Gracia Adolfo^3^, Sanchez-Flores Alejandro*^1^ and Pardo-Lopez Liliana*^1^

^1^ Instituto de Biotecnología, Universidad Nacional Autónoma de México, Cuernavaca, Mor, México.

^2^ Departamento de Innovación Biomédica. CICESE. Carretera Ensenada-Tijuana 3918, Zona Playitas, Ensenada, BC, México

^3^ Instituto de Ciencias del Mar y Limnología, Universidad Nacional Autónoma de México, CDMX, México.

^4^ Current address:CONACYT-Instituto de Investigaciones Agropecuarias y Forestales, Universidad Michoacana de San Nicolás de Hidalgo, Morelia, Mich, México

^+^ These authors contributed equally to this work.

*** Correspondence:**

Liliana Pardo-Lopez

liliana@ibt.unam.mx

Alejandro Sanchez-Flores

alexsf@ibt.unam.mx

# Supplementary Figures


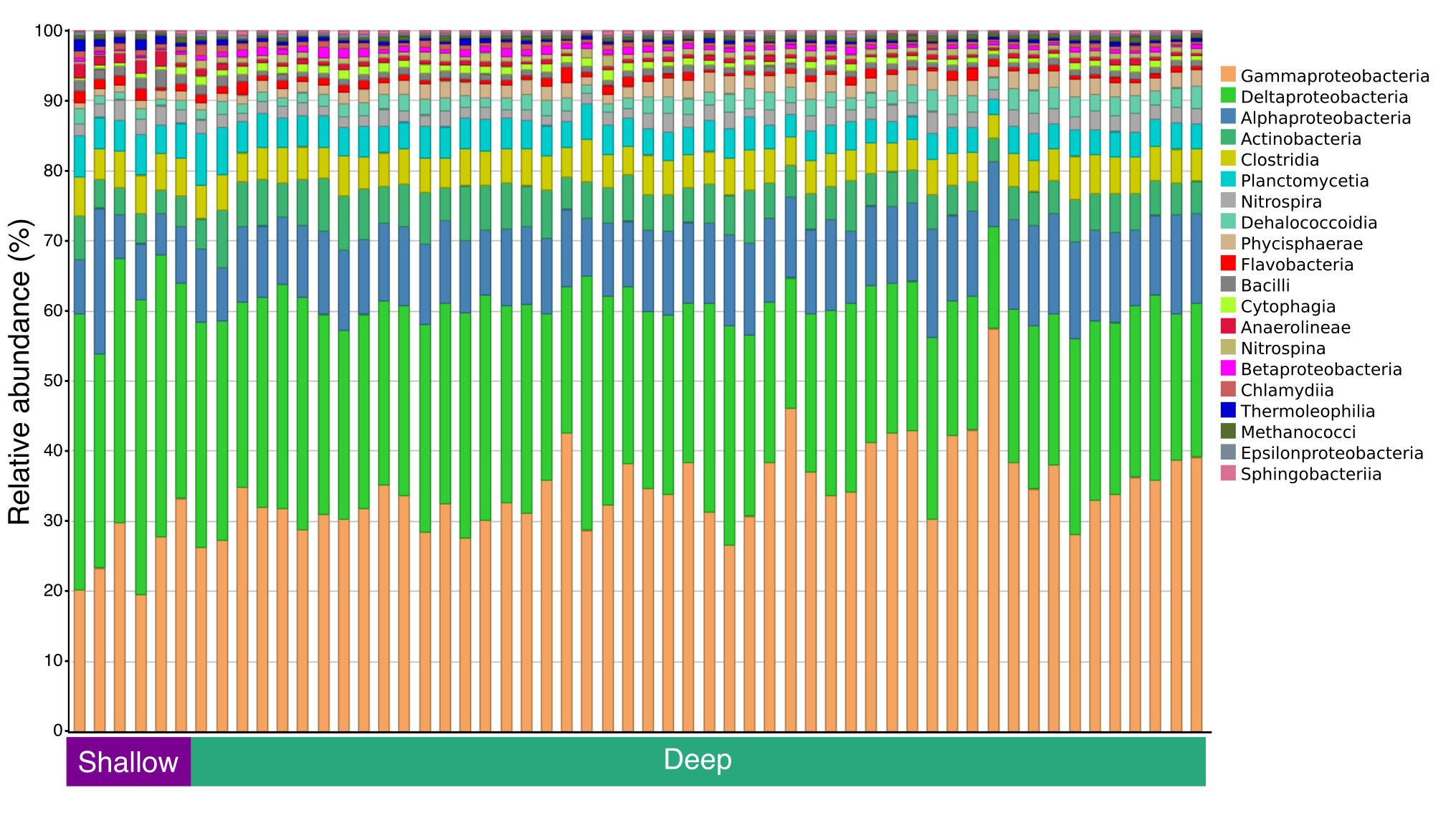


**Supplementary Figure 1. Taxonomic profiles of the most abundant classes in sediment samples from the swGoM.** The profiles are homogeneous, and the dominant classes are *Gamma*, *Delta* and *Alphaproteobacteria* in order of increasing abundance.


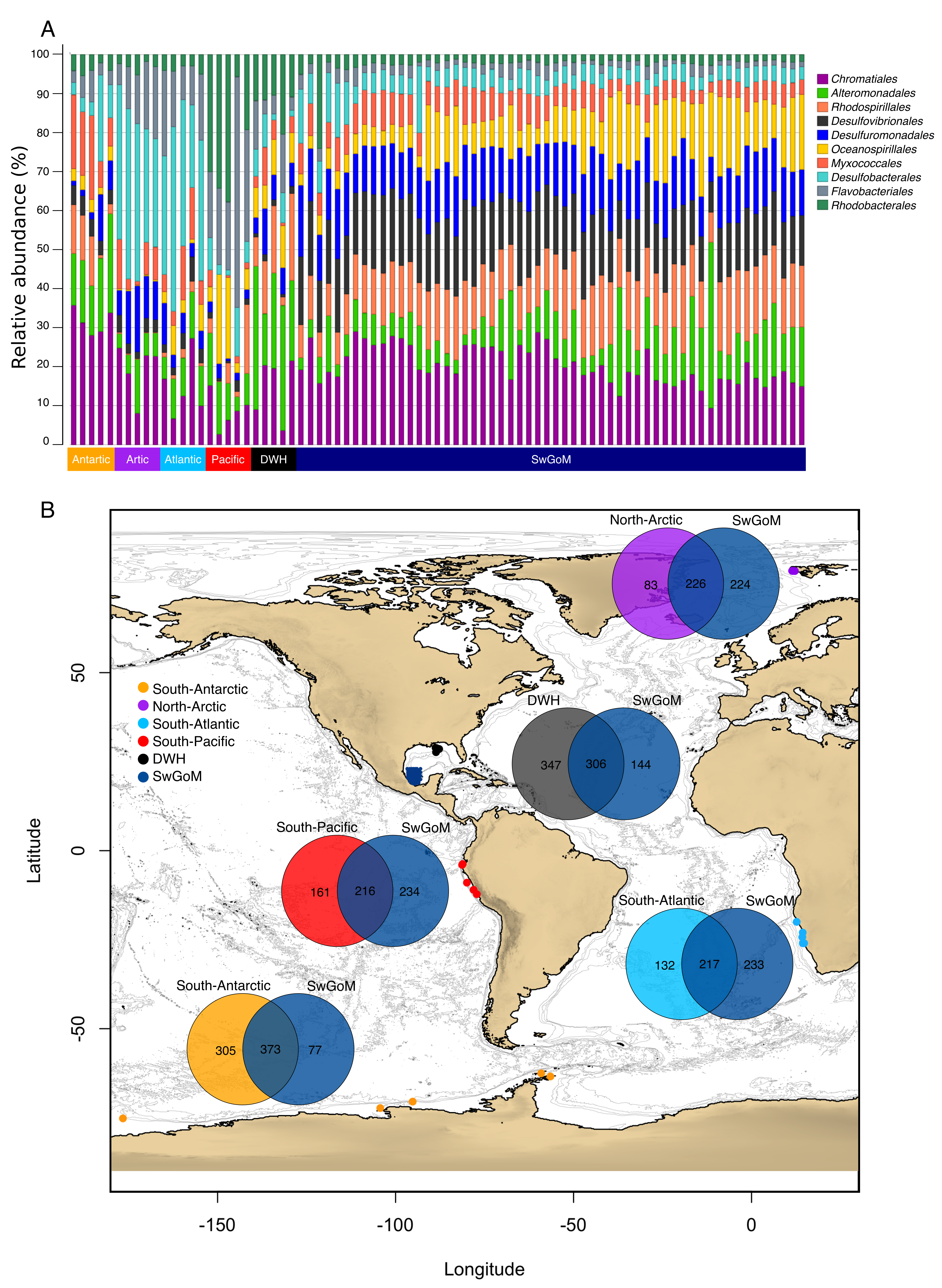


**Supplementary Figure 2. Bacterial diversity comparison between swGoM and sediments from other oceans.** A) Bacterial taxonomic profile of the most abundant orders. B) Geographic location of projects selected for comparison to the swGoM and Venn diagrams representing the genera shared between them.


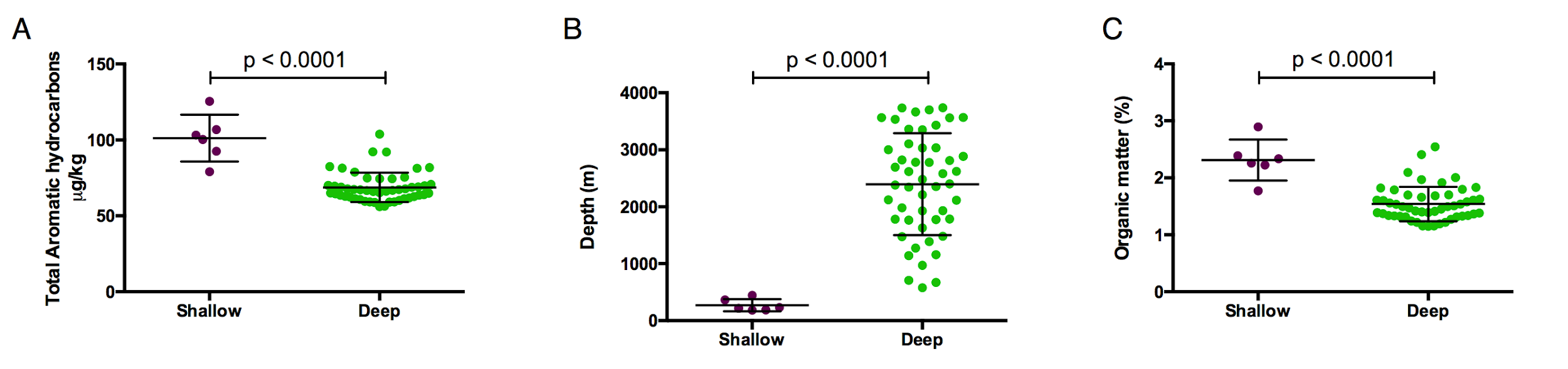


**Supplementary Figure 3. Statistical differences between shallow and deep groups in the functions of abiotic parameters with higher contributions to sample distribution.** A) Total aromatic hydrocarbons, B) depth, and C) organic matter distribution in the shallow and deep groups.


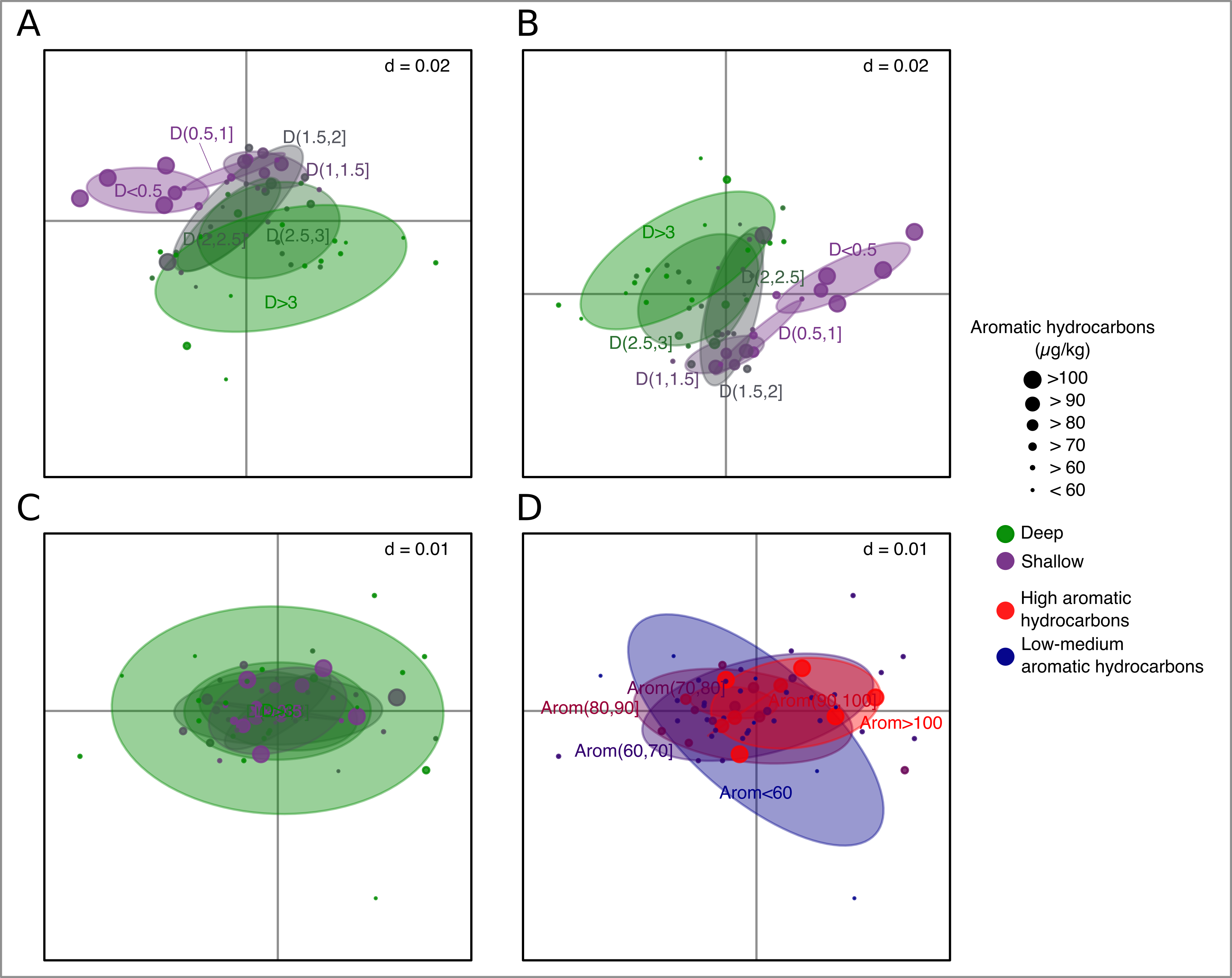


**Supplementary Figure 4. Constrained double principal coordinates analysis (cDPCoA)** integrating phylogenetic distances at the genus level and considering the variables of aromatic hydrocarbon concentration and depth as confounding factors. A) DPCoA incorporating phylogenetic distances of the genus matrix and groups, colored by depth. B) DPCoA highlighting the variance by aromatic hydrocarbons content and groups, colored by depth. C) DPCoA removing the variance produced by depth and colored by depth. D) DPCoA removing the variance produced by depth and groups, colored by aromatic hydrocarbons concentration.
